# Nutritional interventions to augment immunity for COVID-19

**DOI:** 10.1038/s41387-022-00194-3

**Published:** 2022-03-30

**Authors:** Samer Singh, Rakesh K. Singh

**Affiliations:** 1 Centre of Experimental Medicine & Surgery, Institute of Medical Sciences, Banaras Hindu University, Varanasi, 221005 India; 2 Department of Biochemistry, Institute of Science, Banaras Hindu University, Varanasi, 221005 India

**Keywords:** Nutritional supplements, Patient education, Policy and public health in microbiology, Ageing, Risk factors

**Dear Editor,**

Nutrients are critical for immune functions. Calder, P.C. has discussed the key role of good nutrition in immunity against COVID-19 [1]. It emphasizes the need for appropriate nutrition supplementation to people who are elderly, frail, and/or suffering from obesity, diabetes, malnutrition, etc., and have been adversely impacted by COVID-19. We agree with the general message of good nutrition for stronger immunity and mortality reduction, but nutrient supplementation during active infections should be treated cautiously. We feel the aspects of nutritional immunity and metalloimmunology should have been included to avoid the impression of unrestricted nutrient supplementation recommendation, especially of metals during acute infections [2, 3]. It can precipitate adverse outcomes in patients with obesity and diabetes who display chronic inflammation and metal-ions deficiency in serum.

During infections and chronic inflammations, the levels of various metal ions are decreased/altered as a part of ‘nutritional immunity’ to reduce pathogen survival, enhance immunological surveillance and response, and minimize oxidative damage of vital tissues (endothelial integrity, cardiovascular function, etc.) from the heightened inflammatory response [2, 4, 5]. Contrary to their portrayal in recent literature, low serum Zn levels never reflect deficiency, rather they are reflective of individuals’ physiology and the disease status [6, 7]. Supplementation of Zn could effectively reverse the protective anti-oxidant role of Zn into the pro-oxidant, pro-inflammatory and pro-apoptotic, causing increased cytotoxicity, inflammation, and tissue damage [3, 5]. For realizing any anti-viral activity benefit from Zn supplementation, >1–2 orders of magnitude higher [Zn^2+^] is required than that attainable in vivo [8]. Though Zn supplementation augments the immune systems of deficient individuals and reduces their chance of getting an infection, the benefit of supplementation for already sufficient or over supplemented individuals—the majority, remains debatable. The lower serum Zn levels observed for severe COVID-19 in studies quoted in [1] are as expected. The severity of the disease is expected to drive acute phase response reducing the free Zn levels to limit the damages to host tissues from oxidative stress [3, 5]. It may be pertinent here to mention the data available on Zn supplementation of individuals with HIV infection or active tuberculosis (TB)—two comorbid conditions of COVID-19. Zn supplementation had increased the mortality rate in HIV-infected by about three-fold over the standard treatment [9]. In the patients undergoing TB treatment, it delayed clearance of *Mycobacterium tuberculosis* from sputum [10]. In individuals with diabetes, who already have a higher oxidative-stress burden due to high glucose, the Zn supplementation during infections could further aggravate the oxidative damage to tissues by compromising the activity of the ROS-handling system [5]. The populations with higher dietary Zn sufficiency have seen up to 150–200 times more COVID-19 mortality than populations with lower Zn sufficiency [11]. The unnecessary Zn intakes during acute infections could potentially invite fatal complications in patients with Zn dyshomeostasis (e.g., elderly, diabetic) by disturbing the homeostasis and causing increased oxidative-stress and mortality [3, 5].

The unrestricted nutrient supplementation during the acute phase of disease poses potential risks [2–5]. The inclusion of cautionary notes in articles dealing with nutrition for well-being may be considered as they get read by a much larger audience including physicians and policy-makers.
